# Influenza incidence and air pollution: Findings from a four-year surveillance study of prefecture-level cities in China

**DOI:** 10.3389/fpubh.2022.1071229

**Published:** 2022-12-02

**Authors:** Yu Zhang, Shijun Wang, Zhangxian Feng, Yang Song

**Affiliations:** ^1^School of Geographical Sciences, Northeast Normal University, Changchun, China; ^2^Key Laboratory of Geographical Processes and Ecological Security in Changbai Mountains, Ministry of Education, Changchun, China

**Keywords:** influenza incidence, air pollutants, sensitivity, sensitive division, China

## Abstract

**Background:**

Influenza is a serious public health problem, and its prevalence and spread show significant spatiotemporal characteristics. Previous studies have found that air pollutants are linked to an increased risk of influenza. However, the mechanism of influence and the degree of their association have not been determined. This study aimed to determine the influence of the air environment on the spatiotemporal distribution of influenza.

**Methods:**

The kernel density estimation and Getis-Ord Gi^*^ statistic were used to analyze the spatial distribution of the influenza incidence and air pollutants in China. A simple analysis of the correlation between influenza and air pollutants was performed using Spearman's correlation coefficients. A linear regression analysis was performed to examine changes in the influenza incidence in response to air pollutants. The sensitivity of the influenza incidence to changes in air pollutants was evaluated by performing a gray correlation analysis. Lastly, the entropy weight method was used to calculate the weight coefficient of each method and thus the comprehensive sensitivity of influenza incidence to six pollution elements.

**Results:**

The results of the sensitivity analysis using Spearman's correlation coefficients showed the following ranking of the contributions of the air pollutants to the influenza incidence in descending order: SO_2_ >NO_2_ >CO> PM_2.5_ >O_3_ >PM_10_. The sensitivity results obtained from the linear regression analysis revealed the following ranking: CO>NO_2_ >SO_2_ >O_3_ >PM_2.5_ >PM_10._ Lastly, the sensitivity results obtained from the gray correlation analysis showed the following ranking: NO_2_ >CO>PM_10_ >PM_2.5_ >SO_2_ >O_3._ According to the sensitivity score, the study area can be divided into hypersensitive, medium-sensitive, and low-sensitive areas.

**Conclusion:**

The influenza incidence showed a strong spatial correlation and associated sensitivity to changes in concentrations of air pollutants. Hypersensitive areas were mainly located in the southeastern part of northeastern China, the coastal areas of the Yellow River Basin, the Beijing-Tianjin-Hebei region and surrounding areas, and the Yangtze River Delta. The influenza incidence was most sensitive to CO, NO_2_, and SO_2_, with the occurrence of influenza being most likely in areas with elevated concentrations of these three pollutants. Therefore, the formulation of targeted influenza prevention and control strategies tailored for hypersensitive, medium-sensitive, low-sensitive, and insensitive areas are urgently needed.

## Introduction

Influenza poses a serious threat to human health because of its high contagiousness and incidence. Several global pandemics have directly caused casualties and indirectly caused economic losses, which creating major problems for many countries in the last hundred years (1). Influenza is an infectious disease that has not been fully brought under control (2). China's urbanization and ecological civilization construction processes remain to be synchronized, which has indirectly resulted in the integration and complication of influenza-influencing factors and the generation of numerous potential health-related crises within the population (3, 4). Historical experience has shown that widespread epidemics of infectious diseases, such as influenza, are often exacerbated by the polluted air environment (5). Air pollutants are plausible biological factors explaining the occurrence of influenza cases. Short- and long-term exposure to air pollutants increase the risk of morbidity and mortality from a wide range of systemic diseases, including cardiovascular, respiratory, and other diseases, thereby contributing to the emergence and spread of influenza (6, 7). In 2012, the International Council for Science launched the Future Earth Initiative. This initiative emphasizes the need to strengthen research on the direct and complex relationship between changes in environmental pollution and human health. The Chinese government has also proposed the Healthy China Initiative, which meets national priorities and the emphasis on sustainable development. The government has clearly identified measures required to implement comprehensive urban air quality management to meet standards and promote significant improvements in ambient air quality in cities nationwide and effectively resolve outstanding environmental problems that affect the health of the population (8). The following questions arise. What have been the characteristics of the spatiotemporal distribution of influenza and air pollutants in recent years? How can the relationship between the incidence of influenza and air pollutants be studied? How can the pattern of sensitivity of influenza to air pollutants be scientifically measured? These issues are major problems and challenges for the prevention and control of influenza and air pollutants in key areas and regions of their occurrence.

The notion that airborne pollution particles provide “condensation nuclei” to which influenza virus droplets attach has been prevalent within environmental health research for several decades. Toxicological studies have suggested that air pollutants are biologically plausible factors leading to the occurrence of influenza-like cases. The main mechanisms driving these cases include inflammatory responses, oxidative stress, and genetic damage (9–11). Exposure to air pollutants, which produce free radicals, can lead to mucosal irritation of the airways and mechanical damage, affecting mucus clearance by cilia and reducing an individual's resistance to viral infections, such as influenza (12). Epidemiological evidence also suggests that short or long-term exposure to air pollutants significantly increases the risk of influenza morbidity and mortality (13–18). Different pollutants have different health effects on the population (19, 20). PM_2.5_ contains toxic substances, such as nickel, vanadium, acidic oxides, and pathogenic bacteria. When inhaled into the airways, these toxic substances adsorb to the alveoli, where they interact with the surfactant secreted by the lung cells, causing damage to the alveolar walls. This process causes inflammation and increases the vulnerability of the population to viruses (21). PM_10_ contains heavy metals, polycyclic aromatic hydrocarbons, and other toxic and harmful substances, which can lead to lesions in human organs following their entry into the alveoli (22). SO_2_ stimulates peripheral nerve receptors in the smooth muscles of the upper and bronchial airways, weakening the ability of the respiratory tract to block pathogens and inducing susceptibility to infection by the influenza virus (23). Nitrogen oxides can generate irritants, such as HNO_2_ and HNO_3_, when they enter the alveoli through inhalation. This process increases the permeability of lung capillaries and causes respiratory diseases, such as bronchitis, pneumonia, and emphysema, and heightens the risk of influenza infection (24). Inhalation of a certain concentration of CO into the body decreases oxygen absorption into the blood. Moreover, the altered dissociative properties of oxyhemoglobin further reduce oxygen delivery to the tissues, and resistance to the influenza virus is reduced (25). Inhalation of certain concentrations of O_3_ promotes lipid peroxidation in the epithelial cells of the respiratory tract, which increases the production of arachidonic acid. Such substances induce inflammatory lesions in the upper respiratory tract, weakening its defenses (26). Pathogenic reactions of these air pollutants in humans increase the potential for the production and transmission of influenza viruses within the population.

The impacts of six key air pollutants on the incidence of influenza have rarely been studied in the field of environmental health. Applying a generalized summation model, Feng et al. (27) demonstrated that ambient PM_2.5_ concentrations were significantly associated with the risk of influenza-like illness in Beijing during the flu season and that the effect of PM_2.5_ differed across age groups, in this city. Adults comprised the most significantly affected population, probably because of their longer exposure to outdoor pollution. Ali et al. (28) reported a statistically significant negative association between O_3_ and influenza transmission in Hong Kong, China. They suggested that this finding could be related to the virucidal activity of O_3_ and its effect on host defense and even immunity to influenza viruses. Liu et al. (16)found a positive association between PM_2.5_ and the incidence of clinical influenza in Hefei Province in China and a negative association between PM_10_ and the incidence of clinical influenza. No relationship has been reported between NO_2_ concentrations and the influenza incidence. Su et al. (29) found that PM_2.5_, PM_10_, CO, and SO_2_ concentrations were associated with influenza-like cases in Jinan, China. Moreover, there was a lagged effect of air pollutants on the incidence of influenza. Recent studies on the correlation between the influenza incidence and air pollutants have mostly applied statistical models, such as correlation analysis (30), regression analysis (30), machine learning (31), and non-linear models (32). To the best of our knowledge, few studies have developed and applied sensitivity analysis to examine the relationship between disease and environment. Sensitivity analysis is an important research method for measuring changes and interactions among geographic elements (33). This research approach constitutes a frontier and hotspot within geographic environmental modeling research (34). It can be effectively used to identify the main environmental pollutants linked to the influenza incidence and to identify the magnitude of the contribution of each risk factor to the influenza incidence (35–38).

Relatively few studies have quantitatively analyzed the association between influenza and the full range of air pollutants. Most of these studies have been conducted at the level of prefecture-level cities within individual provinces. However, the spatiotemporal distribution of influenza determined through a nationwide study encompassing municipal, macro, meso, and micro scales and a long-time series has not yet been conducted. Relatively few studies have applied sensitivity analysis in the field of health geography. Accordingly, we collected data on the incidence of influenza and air pollutants in Chinese prefecture-level cities during the period 2014–2017 and explored the spatial clustering characteristics of influenza and air pollutants in these cities. Moreover, we studied the interrelationship between infection with human influenza and air environment factors using Spearman's correlation coefficients, a linear regression model, and gray correlation analysis in an attempt to address this research gap. The results obtained were used to delineate national influenza control zones.

This study had three aims. The first was to determine the influence of the air environment on the spatiotemporal distribution of influenza and to enrich the theory informing the relationship between the environment and infectious diseases in health geography. The second was to build a “comprehensive model of susceptibility” to generate hierarchical and progressive innovation in susceptibility research methods and to provide a new research paradigm for exploring the relationship between infectious diseases and environmental pollution. The final aim was to provide theoretical support and policy inputs for national and local Centers for Disease Control and Prevention to implement infectious disease prevention and control, formulate regionalized prevention and control strategies, establish long-term prevention and control mechanisms and set environmental health standards, and effectively contribute to and advance the Healthy China Initiative.

## Materials and methods

### Theoretical framework

**Figure 1:**
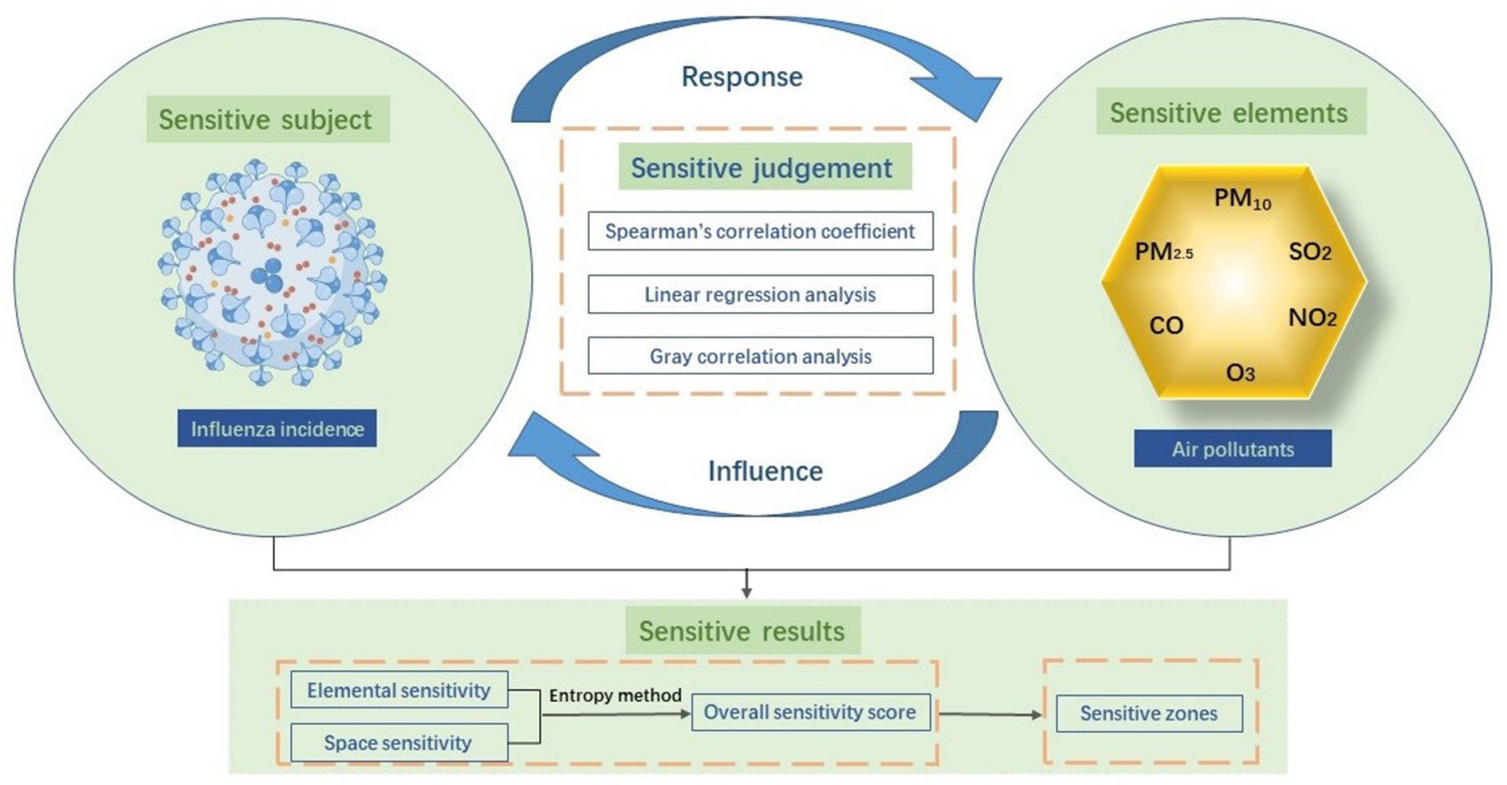
Theoretical framework.

We introduced a sensitivity research framework for investigating the relationship between the influenza incidence and air pollutants. As shown in Figure 1, we applied a method for characterizing the degree of response of one factor to a change in another factor. Our framework, which encompassed sensitive subjects, factors, judgments, and results, was aimed at discovering scientific laws of organic correlation between two or more factors. A sensitive subject was defined as a system or individual whose response is affected by a change in one or more factors. In the context of environmental health, sensitive subjects include the status of human health, disease morbidity, and mortality. In this study, sensitive subjects mainly referred to the influenza incidence. Sensitive elements were defined as factors that lead to the changes that influence sensitive subjects and involve the natural environment as well as socioeconomic and individual factors. In our study, these elements were the six air pollutants elements: PM_2.5_, PM_10_, SO_2_, NO_2_, CO, and O_3_. Changes in sensitive elements affect sensitive subjects, which consequently respond to these elements.

To assess sensitivity, we performed a Spearman's correlation analysis, linear regression analysis, and gray correlation analysis, which could determine the specific impacts of sensitive elements on subjects. Spearman's correlation coefficients were applied to determine a simple correlation between the influenza incidence and air pollutants, while regression analysis was performed to observe their fitting effect according to their level of correlation, and gray correlation analysis was performed to assess the degree of association. In light of an overview of the results of each model, we ranked elemental sensitivity and spatial sensitivity to morbidity for each of the pollutants. Accordingly, we delineated prefecture-level cities into high, medium, low, and insensitive zones according to their sensitivity scores. This research paradigm can be extended to cover more disease types and research areas and provides a theoretical and methodological foundation for tracing environmental pollution and health protection technologies relating to diseases of high prevalence at a regional level.

### Data and processing

**Figure 2:**
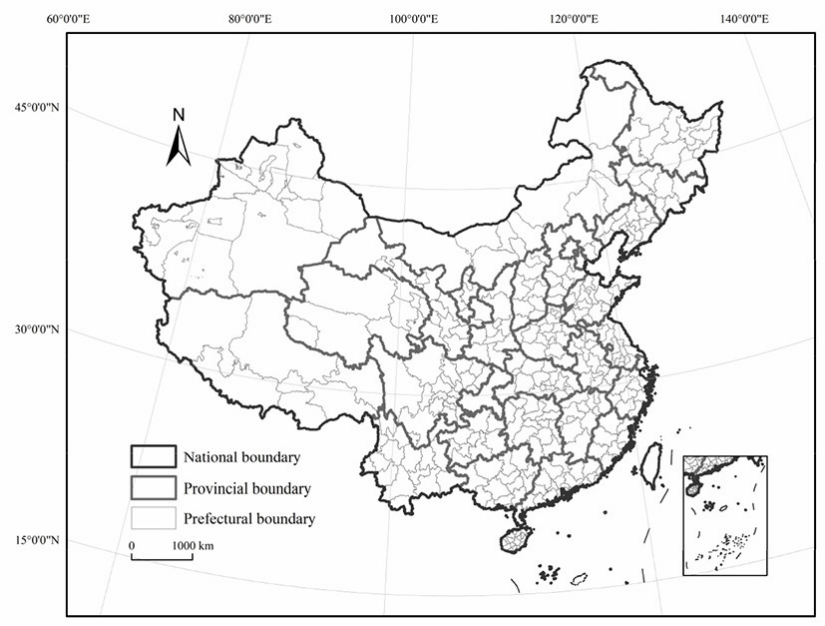
The study area.

Prefecture-level cities constituted the basic unit and scale for this study. We used data compiled from 31 provinces, municipalities, and autonomous regions in China (excluding Hong Kong, Macau, and Taiwan) (Figure 2). The research was conducted from 2014 to 2017, and the compiled data comprised the influenza incidence, air quality, and maps (Table 1). We also obtained monthly data on the influenza incidence in prefecture-level cities. To examine the regional and temporal aspects of the influenza incidence, all of the reported data were utilized as samples. During the study period, the number of cities for which annual data on the influenza incidence were available increased from 359 in 2014 to 366 in 2017. The data were sourced from the Data Center of China Public Health Science, under the China Center for Disease Control and Prevention (https://www.chinacdc.cn/). Given the small number of deaths recorded, we used data on the number of influenza cases calculated as follows: incidence = the number of cases×100 000/ total population. Statistical data for some regions were missing for several years within the study period. Consequently, the data on influenza were not spatially continuous. The missing data were treated as blank and not interpolated. The layers of data used in administrative maps of prefecture-level cities were obtained from the Geographic Information Bureau of the State Bureau of Surveying and Mapping (http://bzdt.ch.mnr.gov.cn/). Pollutant indices for air quality were obtained from real-time air quality monitoring data published by the China National Environmental Monitoring Center (http://www.cnemc.cn/) and included data on fine particulate matter (PM_2.5_), inhalable particulate matter (PM_10_), sulfur dioxide (SO_2_), nitrogen dioxide (NO_2_), ozone (O_3_), and carbon monoxide (CO). A total of 190 cities were monitored daily for air quality in 2014, and 366 were monitored in 2015–2017. Accordingly, the monthly mean value for each city was calculated based on the daily data. Differences in the number of samples between years affected statistical efficiency but did not compromise our statistical inferences because the total coverage was sufficiently large. We have ensured the consistency of the spatial and temporal resolution of the two data through processing. Spatially, because the influenza incidence and air pollution base data were at the prefecture-level city scale and the number of reported prefectures varied among years, we selected the intersection of the two datasets from 2014–2017 as the original dataset for sensitivity analysis, and missing data were treated as blank. Temporally, we calculated monthly means for each city based on daily data of air pollution, to match monthly values of influenza incidence.

**Table 1:** Data source and processing.

**Data**	**Source**	**Processing**
Influenza incidence	The Data Center of China Public Health Science (https://www.chinacdc.cn/)	Incidence = the number of cases × 100 000 people without interpolation
Air quality	The China National Environmental Monitoring Center (http://www.cnemc.cn/)	Calculate monthly mean value by daily data
Maps	The Geographic Information Bureau of the State Bureau of Surveying and Mapping (http://bzdt.ch.mnr.gov.cn/)	Processed and produced by the standard map with the review number GS (2019)1825, with no modification

### Methods

#### Spatial analysis

Kernel density estimation is a non-parametric method of estimating the probability density function of a random variable (39). The method is particularly useful for analyzing and displaying the distribution of influenza incidence.

The local Getis-Ord Gi^*^ hotspot detection is a typical statistical method of local spatial autocorrelation that can be used to identify spatial variation (40). It can accurately reflect the distribution of hotspots for air pollutants in a given area.

#### Statistical analysis

Correlations between air pollutant concentrations and influenza incidence during the study period were estimated using Spearman's correlation coefficients due to the abnormal distributions of all these variables (41).

Regression analysis is a statistical analysis method used to determine the quantitative relationship of interdependence between two or more variables. A linear regression model of the influenza incidence and air pollutants was developed to enable the degree of the sensitivity to the influenza incidence to changes in air pollutants to be quantitatively analyzed.

Gray correlation analysis is performed to measure the closeness of association by comparing the geometric similarity of curves composed of multiple series (42). This method of statistical analysis has no rigid requirements on the sample size and on the presence (or not) of a pattern connecting samples. It was deemed a useful method for conducting a more in-depth evaluation of the sensitivity of the influenza incidence to changes in air pollutants as the sensitive elements. The first step entailed processing the index data indicator data without considering their dimensions. Next, the correlation coefficient was calculated, with the discriminant coefficient, ρ ϵ [0, 1], used to weaken the effect of distortion induced by excessive maximum values. The value of ρ is usually taken as 0.5. The third step entailed calculating the correlation; a higher correlation corresponded to a stronger association between air pollutants and the influenza incidence. According to previous studies (43), the correlations can be classified into four levels. When 0 < *r*_*ij*_≤ 0.35, the correlation is weak; when 0.35 < *r*_*ij*_ ≤ 0.65, the correlation is moderate; when 0.65 < *r*_*ij*_≤ 0.85, the correlation is strong; when 0.85 < *r*_*ij*_≤1.00, the correlation is extremely strong.

The concept of entropy originates from thermodynamics and is a measure of the uncertainty of the state of a system. The entropy weight method is a mathematical method used to judge the degree of dispersion of a certain index, enabling the index to be assigned and calculated more objectively. A larger value indicates that more information provided by the index corresponds to its stronger influence on the comprehensive evaluation, and to a higher associated weight. We used the entropy weighting method to compare and synthesize the sensitivity results obtained using different methods. The detailed procedures of entropy method are described as follows (44):

(1) Standardize the original value of indicators:
(1) $$\begin{array}{l} X_{ij}^{\prime }=\frac{X_{ij}-\min(X_{j})}{\max(X_{j})-\min(X_{j})} \end{array}$$
  where $X_{ij}^{\prime }$is the standardized value of the *i*th evaluating object on the *j*th indicator, *X*_*ij*_ is the original value, and the original value of this study mainly refers to the absolute value of the median of the correlation coefficient, the absolute value of the mean of the regression coefficient and the absolute value of the gray correlation coefficient; max(*X*_*j*_) and min(*X*_*j*_) are the maximum and the minimum, respectively.
(2) The proportion of the *i*th evaluating object on the *j*th indicator is calculated:
(2) $$\begin{array}{l} Y_{ij}=\frac{X_{ij}^{\prime }}{{\sum^{\;}}_{i=1}^{m}X_{ij}^{\prime }} \end{array}$$
  where *m* is the number of evaluating objects, which refers to six air pollutants in this study.
(3) The entropy of each evaluating indicator can be defined as:
(3) $$\begin{array}{l} e_{j}=-k\overset{m}{\underset{i=1}{\sum }}\left(Y_{ij}\times \ln\;Y_{ij}\right) \end{array}$$
  Where $k=\frac{1}{\ln\;m}$. The evaluating object *i* on the indicator *j* is excluded if *Y*_*ij*_ = 0.
(4) The redundancy of the entropy is computed as follows:
(4) $$\begin{array}{l} d_{j}=1-e_{j} \end{array}$$
(5) The weight of entropy of each evaluating indicator could be expressed as:
(5) $$\begin{array}{l} w_{j}=\frac{d_{j}}{\sum_{j=1}^{n}d_{j}} \end{array}$$
  where *n* is the number of methods in this study.

Weights for all methods are listed in Table 2.

**Table 2:** The weights for each method.

**Method**	**Weight**
Spearman's correlation method	0.14
Regression analysis	0.77
Gray Correlation analysis	0.09

The comprehensive sensitivity score was calculated using the weights obtained with the entropy weighting method, which were then multiplied by the standardized data of each indicator to obtain its sensitivity score. Next, the sensitivity scores of each indicator for each prefecture-level city were summed. The calculation formula was as follows:


(6) $$\begin{array}{l} S_{i}=\overset{6}{\underset{n=1}{\sum }}\left(C_{n}\times 0.14+R_{n}^{*}0.77+G_{n}^{*}0.09\right) \end{array}$$

where *S*_*i*_ denotes the sensitivity composite score for prefecture-level cities; *i* is the prefecture-level city; *C*_*n*_ denotes normalized data for correlation coefficients between morbidity and six air pollutants in the Spearman's correlation analysis; *R*_*n*_ denotes normalized data for the correlation coefficient between morbidity and six air pollutants in the linear regression model; *and G*_*n*_ denotes normalized data for the correlation coefficient between morbidity and six air pollutants in the gray correlation analysis.

## Results

### Analysis of the pattern of the influenza incidence and air pollutants

**Figure 3:**
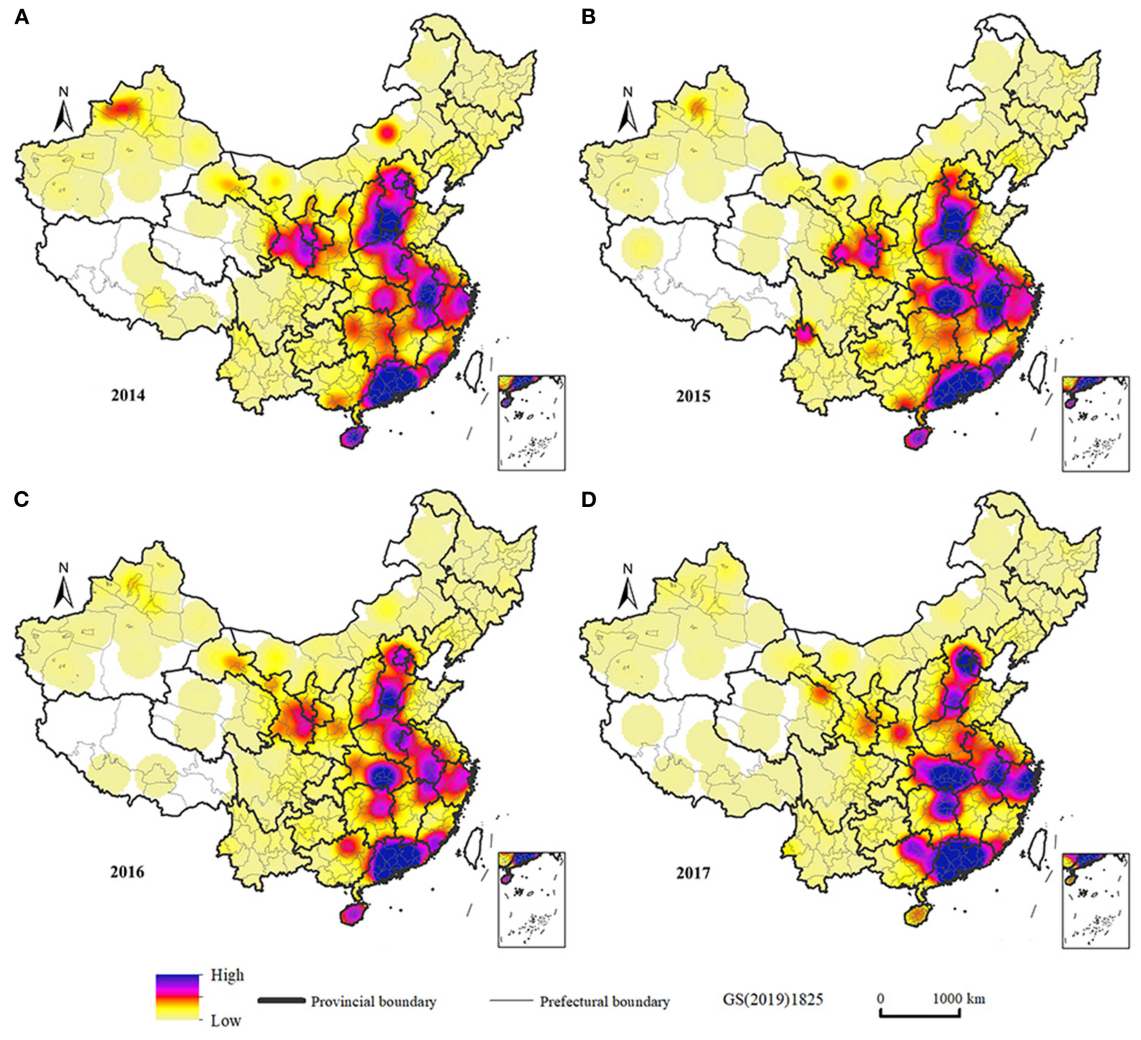
Kernel density estimation of influenza incidence in China. **(A)** Influenza incidence in 2014. **(B)** Influenza incidence in 2015. **(C)** Influenza incidence in 2016. **(D)** Influenza incidence in 2017.

We analyzed the kernel density of influenza incidence in the study area, and hotspot agglomeration area could be judged from the peak distribution of kernel density values. Relevant studies have shown that the larger the search radius, the smoother the surface of the generated results, and the selection of the search radius value also changes with the scale of the study (45). In this study, we examined the distribution of agglomerations based on the scale of countries, and thus a high search radius should be chosen. By trying different search radius, the results showed that the spatial difference in density of elements was most obvious at a search radius of 200 km, which portrayed large and medium agglomerations while also outlining relatively small agglomerations. Figure 3 shows the kernel density of the spatial distribution of the influenza incidence in China from 2014 to 2017. The incidence of influenza was high and concentrated in Beijing and the provinces of Hubei, Anhui, Zhejiang, and Guangdong. Zhuhai, Zhongshan, Huizhou, Guangzhou, and Handan were typical high-incidence cities, which may be related to rapid economic development and the increase in the number of factories in recent years.

**Figure 4:**
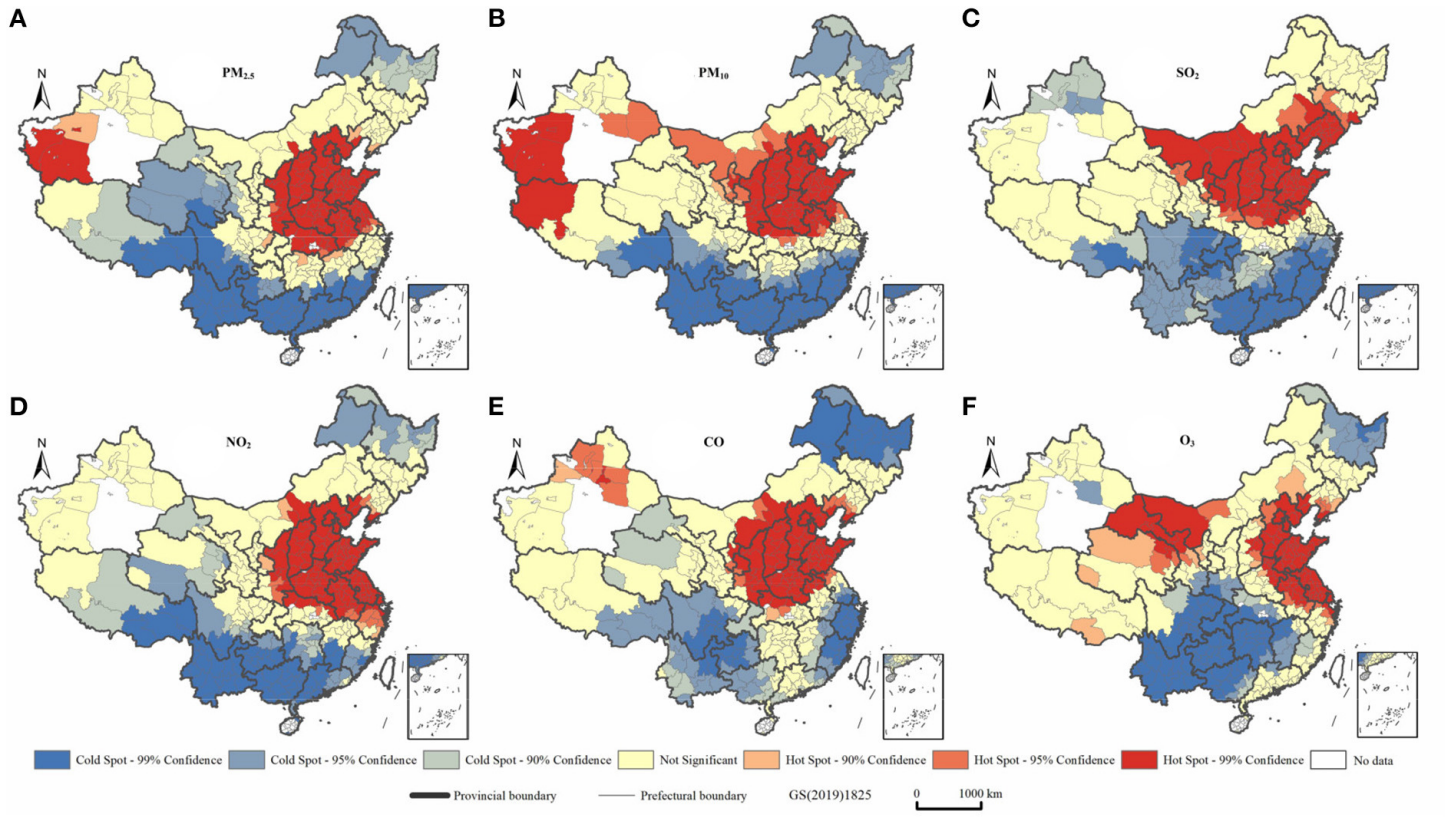
Analysis of six types of air pollutants in China during the period 2014-2017 using the local Getis-Ord Gi^*^ statistic. **(A)** PM_2.5_. **(B)** PM_10_. **(C)** SO_2_. **(D)** NO_2_. **(E)** CO. **(F)** O_3_.

Figure 4 shows the local spatial autocorrelation characteristics of air pollutants. During the period 2014–2017, air pollutants in China showed obvious clustering characteristics and a clear delineation of cold and hot spots. Their distribution characteristics were similar to those of China's population as indicated by the characteristic “Hu line,” indicating the density of distribution of the country's population, which accords with the findings of previous studies (46, 47). Air pollutants tend to cluster in the region east of the Hu line, especially in the Beijing-Tianjin-Hebei region. High-concentration agglomerations were evident in Henan, Hunan, and Shandong Provinces, and at the junction of Shandong and Jiangsu Provinces, which formed hubs from which the air pollutants diffused widely into surrounding areas. The distribution of air pollutants also showed some correlation with topographic features. Specifically, the air pollutants showed significant clustering characteristics in the Tarim and Sichuan Basins and the Kuan-chung, Fenhe, North China, and Yangtze (middle and lower) Plains. These characteristics are associated with the convergence of air pollutant particles, and consequently their limited diffusion, when they are deposited in low-lying terrains (48–52). Some provincial capitals, such as Zhengzhou, Shijiazhuang, Xi'an, Wuhan, Jinan, and Chengdu, are heavily polluted. There has been a trend of expansion to surrounding grades, with these cities at the center. This trend may be related to the per capita car ownership in the provincial capitals and the development of secondary industries (53).

### A sensitivity analysis based on spearman's correlation coefficients

Table 3 shows the correlation coefficients between the influenza incidence and air pollutants in various prefecture-level cities in China, with a *p*-value below 0.05. The correlation coefficients between the respective concentrations of PM_2.5_, PM_10_, SO_2_, NO_2_, CO, and O_3_, and the influenza incidence were all above 0.5. The first five factors had positive values, with the maximum and minimum values within a range of 0.5–1.0 and median values mostly concentrated around a value of 0.75, indicating a strong correlation. However, the correlation coefficients between the O_3_ concentrations and influenza incidence had negative values, with a median value concentrated around −0.750, indicating a negative correlation. Our comparison of the degrees of correlation between the five other air pollutants, which were positively correlated with the influenza incidence, revealed that their absolute values showed differences that were not significant. The correlation between the influenza incidence and the SO_2_ concentration was the highest among all of the pollutants, with the highest median value (0.795). The average median values of NO_2_ and CO concentrations were, respectively, 0.790 and 0.774. Those of PM_2.5_ and PM_10_ were lower, at 0.764 and 0.747, respectively. The average median correlation coefficient between the O_3_ concentration and the influenza incidence was −0.763, indicating a significant negative correlation.

**Table 3:** Median correlation coefficients between the influenza incidence and air pollutants in China's prefecture-level cities during the period 2014–2017.

	**PM_2.5_**	**PM_10_**	**SO_2_**	**NO_2_**	**CO**	**O_3_**
2014	0.769	0.728	0.811	0.810	0.804	−0.851
2015	0.748	0.741	0.769	0.770	0.777	−0.734
2016	0.753	0.765	0.803	0.772	0.752	−0.710
2017	0.785	0.754	0.797	0.809	0.763	−0.754
Mean value	0.764	0.747	0.795	0.790	0.774	−0.762

### A sensitivity analysis based on a linear regression model

**Figure 5:**
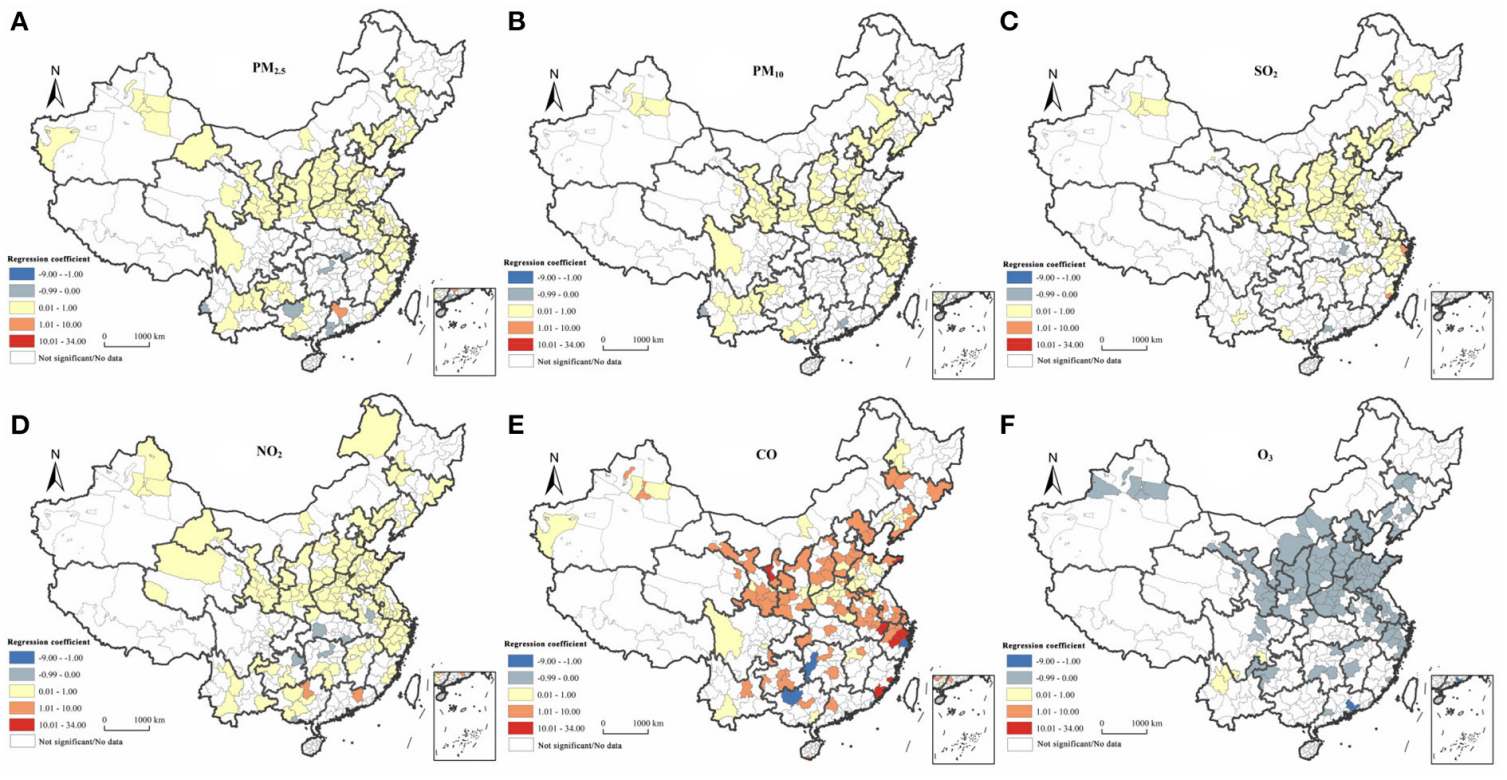
The spatial distribution of regression coefficients between the influenza incidence and six types of air pollutants in China during the period 2014-2017. **(A)** PM_2.5_. **(B)** PM_10_. **(C)** SO_2_. **(D)** NO_2_. **(E)** CO. **(F)** O_3_.

**Figure 6:**
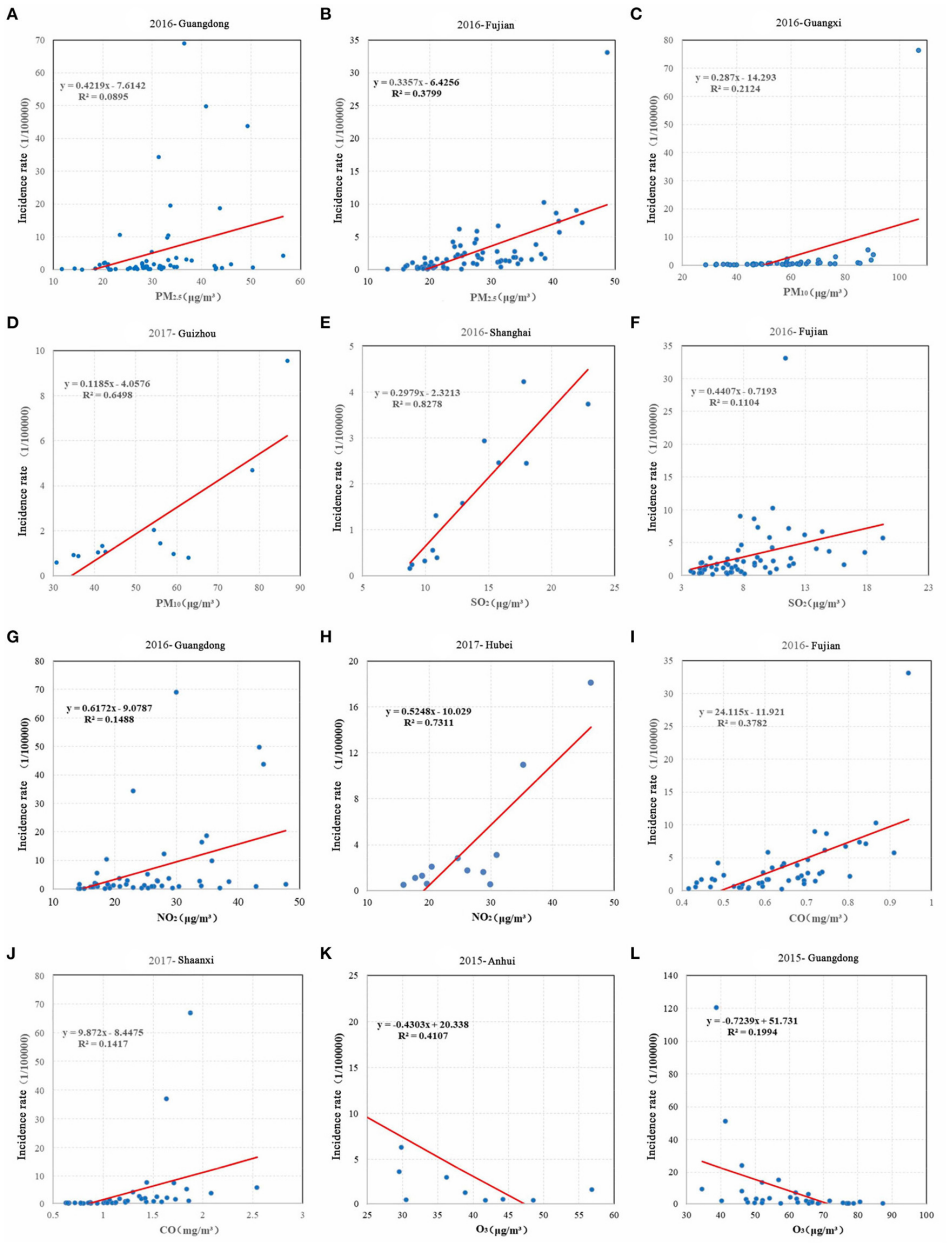
Scatter fitting of the influenza incidence and air pollution in typical sensitive provinces during 2014-2017. **(A)** Regression fitting of PM_2.5_ and incidence rates in Guangdong Province in 2016. **(B)** Regression fitting of PM_2.5_ and incidence rates in Fujian Province in 2016. **(C)** Regression fitting of PM_10_ and incidence rates in Guangxi Province in 2016. **(D)** Regression fitting of PM_10_ and incidence rates in Guizhou Province in 2017. **(E)** Regression fitting of SO_2_ and incidence rates in Shanghai Province in 2016. **(F)** Regression fitting of SO_2_ and incidence rates in Fujian Province in 2016. **(G)** Regression fitting of NO_2_ and incidence rates in Guangdong Province in 2016. **(H)** Regression fitting of NO_2_ and incidence rates in Hebei Province in 2017. **(I)** Regression fitting of CO and incidence rates in Fujian Province in 2016. **(J)** Regression fitting of CO and incidence rates in Shaanxi Province in 2017. **(K)** Regression fitting of O3 and incidence rates in Anhui Province in 2015. **(L)** Regression fitting of O_3_ and incidence rates in Guangdong Province in 2015.

According to the results of the correlation analysis, the prefecture-level cities that showed a strong correlation between the influenza incidence and air pollutants were selected for further analysis to control for the influence of meteorological, topographical, and human factors on the model. Using monthly data on the incidence of influenza and air pollutants in each prefecture-level city from 2014 to 2017, and after screening as the original data, we built a linear regression model to calculate the regression coefficients at the national scale. Figure 5 shows a significant relationship between the influenza incidence and the concentration of air pollutants in the majority of regions. The regression coefficients of PM_2.5_, PM_10_, SO_2_, NO_2_, and CO with the influenza incidence were mostly positive, and the regression coefficients of O_3_ with the influenza incidence were mostly negative. Our results indicated that the influenza incidence was most sensitive to changes in CO concentrations; for example, for every 1 μg/m3 increase in the CO concentration in Ningbo, the influenza incidence increased by 32.54. For every 1 μg/m3 increase in the CO concentration in Zhangzhou, the influenza incidence increased by 33.18. The ranking of the contribution of each element to the influenza incidence, in descending order, was as follows: CO>NO_2_ >SO_2_ >O_3_ >PM_2.5_ >PM_10_. Areas demonstrating significant sensitivity were mostly located in the Beijing-Tianjin-Hebei region; the Fenwei Plain, Jiangsu, Anhui, Shandong, Henan and the Yangtze River Delta. Referring to the regression results for the influenza incidence and the six air pollutants during the period 2014–2017, we selected provinces in which the influenza incidence was more sensitive to changes in air pollutants for a scattering fitting. Figure 6 shows a high R^2^ and a good linear fit. Specifically, the influenza incidences in Guangdong, Fujian, Guangxi, and Shanghai were strongly correlated with air pollutants, and the degree of the fit was higher.

### A sensitivity analysis based on gray correlation analysis

We performed gray correlation analysis to calculate the integrated gray correlations between air pollutants and the influenza incidence in prefecture-level cities from 2014 to 2017. Our calculations indicated that the integrated correlations of the influenza incidence with PM_2.5_, PM_10_, SO_2_, NO_2_, CO, and O_3_ concentrations in China were 0.792, 0.793, 0.789, 0.798, 0.796 and 0.753, respectively, and the overall ranking was NO_2_ >CO>PM_10_ >PM_2.5_ >SO_2_ >O_3._ It can be seen that the absolute difference between the influenza incidence and the six air pollutants in China was small, with all of the elements showing a strong correlation with the influenza incidence. The highest correlation occurred between NO_2_ concentrations and the influenza incidence, revealing that they were most strongly related, whereas O_3_ concentrations had a relatively small effect on the influenza incidence.

**Figure 7:**
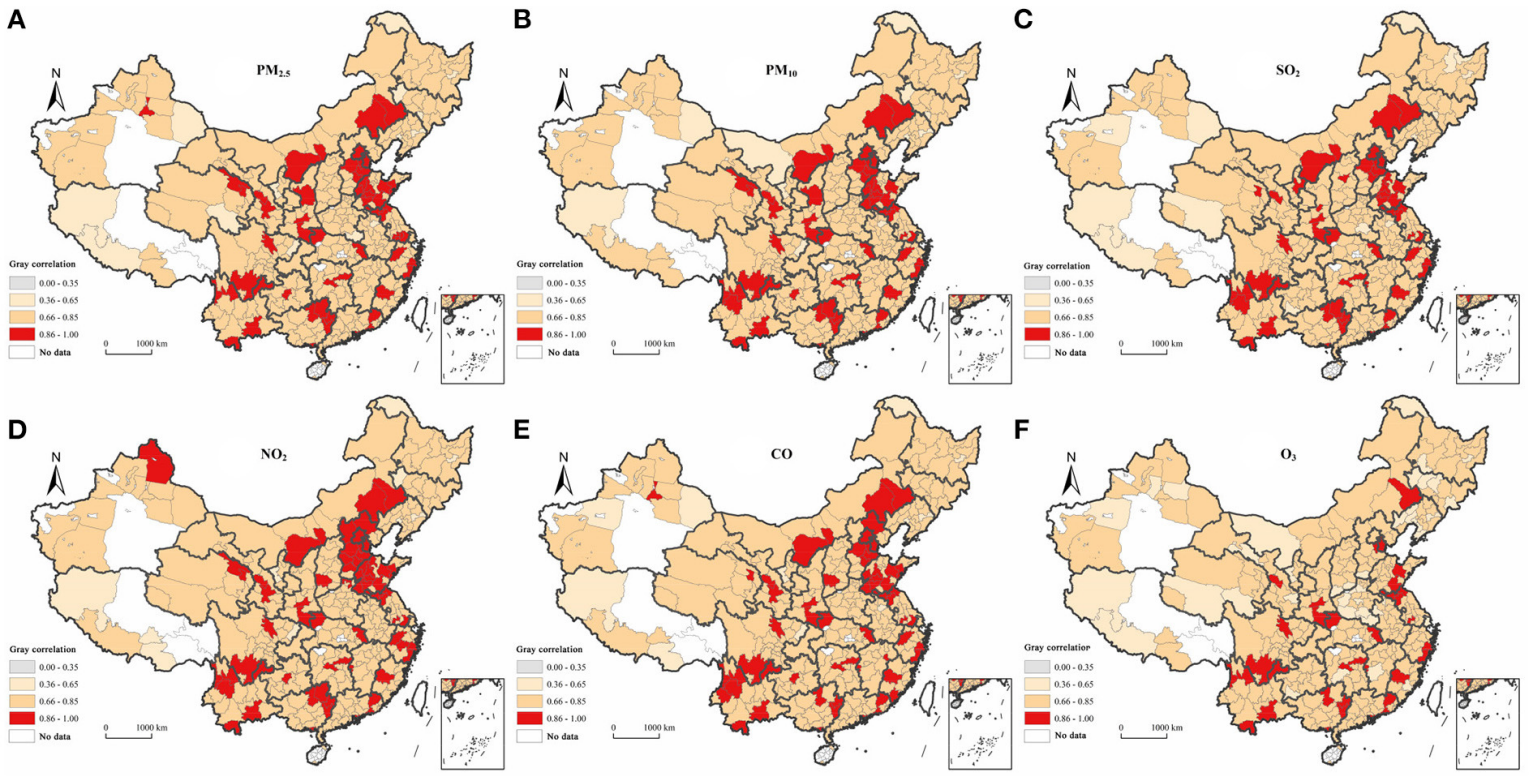
The spatial distribution of the degree of gray association between the influenza incidence and six types of air pollutants in China during the period 2014-2017. **(A)** PM_2.5_. **(B)** PM_10_. **(C)** SO_2_. **(D)** NO_2_. **(E)** CO. **(F)** O_3_.

We averaged the values for the prefecture-level city correlations during the period 2014–2017 and calculated the sensitivity of the influenza incidence to air pollutants in prefecture-level cities. Figure 7 shows that there was a significant geographical correlation between the influenza incidence and air pollutants in China. The degree of correlation relating to the distribution characteristics of each element was relatively consistent, with a large proportion of cities evidencing moderate or stronger correlations. Cities with very strong correlation levels were mainly located in the Beijing-Tianjin-Hebei region, the Yangtze River Delta, and the eastern part of the southwestern region. The strong correlation between NO_2_ concentrations and the influenza incidence occurred in the largest number of cities (320), and conversely, the smallest number of cities (288) evidenced strong correlations between levels of O_3_ and the influenza incidence. This finding is consistent with the results of the sensitivity ranking of sub-elements using gray correlation analysis.

### Results synthesis and sensitive division

The entropy weighting method was performed to determine the weights of three methods (integrated Spearman's correlation coefficients, linear regression analysis, and gray correlation analysis, and Table 2). We applied formula (6) to measure the combined sensitivity and spatial divergence results (Table 4). The sensitivity scores for CO, NO_2_ and SO_2_ were the highest, and the influenza incidence was most sensitive to changes in the concentrations of these three pollutants, with influenza most likely to occur when their concentrations increased.

**Table 4:** The overall score for each index obtained using the entropy weighting method.

**Elements**	**Correlation coefficient**	**Regression coefficient**	**Gray correlation coefficient**	**Overall score**
PM_2.5_	0.35	0.00	0.87	0.13
PM_10_	0.00	0.00	0.89	0.08
SO_2_	1.00	0.01	0.80	0.22
NO_2_	0.90	0.03	1.00	0.24
CO	0.56	1.00	0.96	0.93
O_3_	0.33	0.01	0.00	0.05

**Figure 8:**
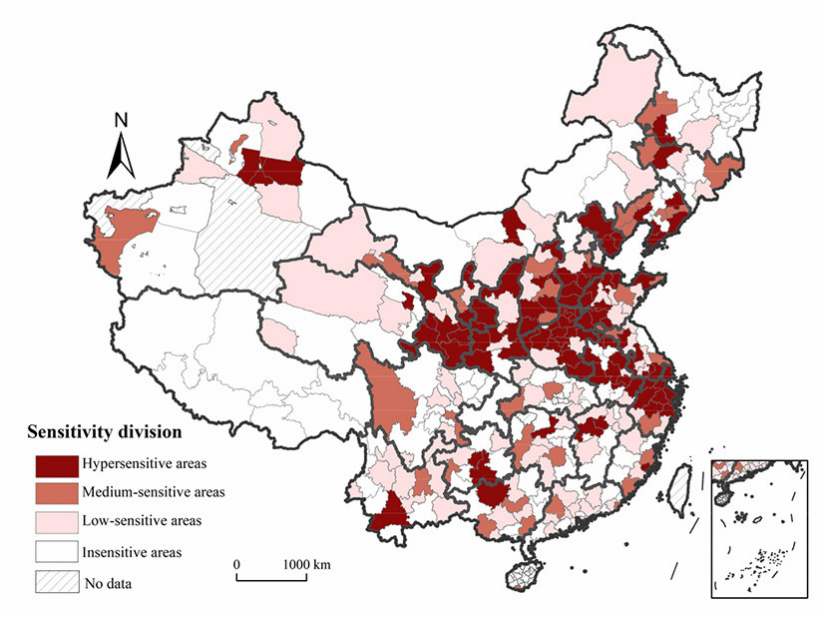
Sensitivity-based zones relating to the influenza incidence and air pollution in China.

Using the natural breaks classification method, we delineated the scores for the prefecture-level cities into three levels (0, 0.60], [0.61, 1.23], and [1.24, 1.67], which respectively corresponded to hypersensitive, medium-sensitive, and low-sensitive areas. Figure 8 shows that the influenza incidence was sensitive to changes in the concentrations of air pollutants in most regions of China. Hypersensitive areas were mainly located in the southeastern part of northeastern China, the coastal areas of the Yellow River Basin, the Beijing-Tianjin-Hebei region and surrounding areas, and the Yangtze River Delta, revealing a “Y” shaped distribution. Influenza is most likely to occur when air pollutant levels rise in these areas. The impacts of changes in the concentration of air pollutants on the influenza incidence in medium-sensitive areas, such as eastern coastal areas, Guangxi and Guizhou Provinces, were not strong. Lastly, in low-sensitive and non-sensitive areas, the impacts of changes in the concentration of air pollutants on the influenza incidence were weaker and more dispersed.

## Discussion

### Discussion of the data

Several weaknesses should be acknowledged in our data. First, the data on influenza used in this study were sampled data, and entailed a certain rate of under-reporting, which influenced the results to some extent (54). Second, we observed differences in statistical capacities across regions and during different years as well as missing data for some periods or regions because of adjustments made in administrative divisions. These problems may have led to deviations between the results obtained and the empirical conditions. Third, the results of the sensitivity analysis of the influenza incidence in relation to air pollutants were correlated with the quantity and quality of the data. Large cities not only have large populations and infected persons, but they also have strong and accurate statistical and reporting capacities. Consequently, the results of the analyses of cities for which more basic data were available tended to be more accurate. But the accuracy of the study results can support the national study due to the sufficient amount of data.

### Discussion of the results

Our findings point to a spatial interpretation of influenza, and are closely aligned with those of previous epidemiological and pathological studies (22, 25, 26, 55–62). Most of the other research results indicated that there was a certain correlation between exposure to air pollutants and disease. When pollutants are absorbed into the blood and tissues, they will have a greater impact on the defense function of the respiratory tract in humans, inducing an inflammatory response in the airways, which could also cause a decrease in the levels of interferon and hemagglutinin inhibitors. These are possible biological mechanisms that increase the risk of influenza-like cases associated with air pollutants (63).

In this study, we observed that the influenza incidence was most sensitive to SO_2_, CO and NO_2_. The underlying mechanism could be that SO_2_ stimulates peripheral nerve receptors in the smooth muscles of the upper and bronchial upper airways, weakening their blockage by the respiratory tract and thus predisposing them to influenza virus infection (23). Inhalation of a certain concentration of CO will reduce the capacity of the blood to absorb oxygen, while changes in the dissociative properties of oxyhemoglobin further reduce the delivery of oxygen to tissues. Consequently, the body's ability to exchange pollutants with oxygen becomes weaker, making it more susceptible to infection by the influenza virus (25). When nitrogen oxides enter the alveoli, they can form irritants, such as HNO_2_ and HNO_3_, which can cause a variety of respiratory diseases, thus increasing the risk of contracting influenza (24). In addition, PM_2.5_ and PM_10_, which are small-sized atmospheric particulate matter, facilitate the attachment of viral droplets to condensation nuclei, thus facilitating the long-range transmission of influenza viruses (64).

The results of this study show that influenza is negatively correlated with the concentration of O_3_ and may have a specific correlation with the bactericidal characteristics of O_3_. However, epidemiological studies indicate that a negative correlation cannot verify the effect of the interaction between the two entities under consideration because they were generated at different times. The concentration of O_3_ tends to be higher in summer, and lower in fall and winter, when a higher incidence of influenza has been observed. Furthermore, long-term ozone inhalation has been associated with increased morbidity and mortality caused by respiratory diseases (65). Moreover, some environmental pollutants may have a lagging effect on human health (66). Therefore, the scientific community should engage in in-depth research to explore the complexity of pathogenic mechanisms.

In addition, we found a large variability in the weights of the three methods using the entropy weighting method, with the regression analysis method weighing 0.77. This may be attributed to the smaller original data for the regression analysis method and the larger standard deviation of the data distribution during the standardization process, resulting in more informative data. According to the definition of the entropy weighting method, the greater the data fluctuation and information, the greater the weight assigned (44). This may slightly affect the accuracy of research results.

### Planning responses and policy suggestions

A novel contribution of this study is its provision of evidence from China that can inform environmental health theories on the relationship between the air environment and human health. Its findings also have important policy implications for guiding the planning and development of healthy cities in China and promoting the construction of a healthy China. Government agencies should consider the following suggestions.

First, we recommend the addition of special planning for influenza and other infectious diseases to the current planning system. Components of this special planning should include establishing a transport system for influenza patients, setting up influenza isolation and treatment facilities, and developing an influenza research system and an early warning system for influenza.

Second, more attention should focus on the Pearl River Delta, the eastern part of China, the Beijing-Tianjin-Hebei region, and other areas with higher incidences of influenza as well as specific cities, such as Beijing, Handan, Zhuhai, Zhongshan, and Huizhou. Efforts should also focus on areas and times of high influenza prevalence.

Third, the distribution of medical institutions should be optimized to facilitate equitable distribution of high-quality medical resources in key high-incidence areas. Additional influenza-specific medical and vaccination points should be set up during the first and fourth quarters of the year when influenza is highly prevalent and people are susceptible to infection.

Fourth, innovations in air-quality eco-compensation programs are needed. A list of enterprises that emit polluting gases, such as SO_2_, CO, and NO_2_, should be developed. More attention should also be paid to the sources of emissions that increase concentrations of PM_2.5_ and PM_10_ (67, 68). The rate of air quality should be established as a binding indicator for atmospheric assessments, and assessments of the weights of SO_2_, CO and NO_2_ concentrations should be conducted more frequently. Furthermore, coefficients of ecological compensation should be optimized, and compensation amounts for curbing the three sensitive pollutant emissions should be increased.

Fifth, in light of the results of our delineation of zones according to the sensitivity of the influenza incidence to changes in the concentrations of air pollutants, we recommend refining prevention and pollution control measures through the formulation of targeted influenza prevention and control strategies tailored for hypersensitive, medium-sensitive, low-sensitive, and insensitive areas. Special guidelines and pollution control should be strategically implemented in hypersensitive areas This strategy encompasses the establishment of county-level special planning for influenza prevention and control and strict control of chemical raw material manufacturing, non-ferrous metal smelting industries, petroleum processing industries, chemical reagent manufacturing, and other industries associated with high CO, SO_2_ and NO_2_ emissions. The location of highly pathogenic industrial spaces close to densely populated areas should be avoided, and low-pollution urban industrial development models should be selected. Efforts should focus on reducing the presence of polluting enterprises with high emissions and high pathogenicity and increasing the introduction and planning of new environmentally friendly industries. A strategy of promoting reasonable layouts and appropriate prevention and control should be pursued in medium-sensitive areas. Specifically, the layout of polluting buildings should be planned reasonably, so that they are located downwind of the city and away from the urban area to reduce the contribution of air pollutants to the influenza incidence. The focus should simultaneously be on the layout of medical buildings, according to the influenza incidence. Prevention-oriented, root-cause prevention and control strategies should be implemented in low-sensitive areas. Accordingly, residents should be actively vaccinated against influenza before the peak influenza season to prevent the emergence and spread of influenza at the source, and possible influenza susceptibility factors in the natural and built environment should be explored.

Lastly, a national influenza risk index should be developed, and the influenza surveillance and early warning system in China should be improved. The six air pollutants should be incorporated as key factors and signals of danger into the influenza classification and warning model, and warning thresholds should be set. Changes in influenza incidence rates, key incidence periods, and key incidence areas under scenarios such as increased pollution and improved pollution should be projected, and early warning programs should be introduced.

### Research limitations

This study had some limitations. First, the effects of the natural and socio-economic factors, such as temperature, precipitation, and population movement, on influenza incidence sensitivity factors were not considered, and the specific causes and effects need to be further explored in the context of other natural and social factors. Second, the lagged effect of influenza on changes in air pollutants was not considered because we used monthly data, whereas the lagged effect of influenza on air pollutants is generally measured in days.

## Conclusion

Influenza outbreaks are a major public health issue worldwide, posing a huge threat to human life and health. We used a top-down approach to analyze the spatiotemporal characteristics of the influenza incidence and air pollutants in China and identified and quantified the relationships between the influenza incidence and parameters of air pollutants within different methodological models. Three main conclusions were derived from this study.

First, high incidences of influenza were concentrated in Beijing as well as in Hubei, Anhui, Zhejiang, and Guangdong Provinces. Air pollutants tended to be concentrated in the area east of the Hu line.

Second, the influenza incidence showed a strong spatial correlation and associated sensitivity to changes in concentrations of air pollutants. Sensitivity was highest in the Yangtze River Delta, the Beijing-Tianjin area, and other areas. The influenza incidence was most sensitive to CO, NO_2_, and SO_2_ levels, with the occurrence of influenza being most likely in areas with elevated concentrations of these three pollutants.

Third, we delineated three sensitivity zones at the national level: hypersensitive, medium-sensitive, and low-sensitive areas according to the combined sensitivity scores obtained for each prefecture-level city. Hypersensitive areas were roughly distributed in a “Y” shaped curve, mainly in the southeastern part of the northeastern China, in coastal areas of the Yellow River Basin, in the Beijing-Tianjin-Hebei region, and in the Yangtze River Delta.

A set of prospects is presented below. First, the scope and sites of research could be appropriately narrowed down in future studies to focus on the association between the micro-scale influenza incidence and air pollutants. Second, more control variables should be incorporated into future studies to explore whether there are any crossover effects of air pollutants with meteorological factors, or with population movements, on the influenza incidence. Third, the impact of the lag effect requires investigation using lagged non-linear models and machine learning. Lastly, it is necessary to construct a comprehensive spatial and temporal risk assessment system and a comprehensive diagnostic model for influenza to assess the environmental risk levels of influenza occurrence nationwide and to develop risk zoning for influenza and other more infectious diseases.

## Data availability statement

The datasets presented in this study can be found in online repositories. The names of the repository/repositories and accession number(s) can be found in the article/supplementary material.

## Ethics statement

The studies involving human participants were reviewed and approved by the Ethics Committee of the Chinese Center for Disease Control and Prevention. Written informed consent to participate in this study was provided by the participants' legal guardian/next of kin.

## Author contributions

YS led the overall study, contributed to the data collection, and interpretation. YZ designed research, analyzed data, and drafted the manuscript. YZ, SW, ZF, and YS contributed to the writing of the manuscript. All authors read and approved the final manuscript.

## Funding

This work was supported by Innovation and Development Strategy Research Project of Science and Technology Department of Jilin Province (20220601022FG), the National Natural Science Foundation of China (42171198 and 42071219), and this work was supported by Data Center of China Public Health Science, part of China Center for Disease Control and Prevention.

## Conflict of interest

The authors declare that the research was conducted in the absence of any commercial or financial relationships that could be construed as a potential conflict of interest.

## Publisher's note

All claims expressed in this article are solely those of the authors and do not necessarily represent those of their affiliated organizations, or those of the publisher, the editors and the reviewers. Any product that may be evaluated in this article, or claim that may be made by its manufacturer, is not guaranteed or endorsed by the publisher.

## References

[1] Yang S Xing X Dong W Li S Zhan Z Wang Q. The spatio-temporal response of influenza A (H1N1) to meteorological factors in Beijing. Acta Geograph Sin. (2018) 73:460–73. 10.11821/dlxb201803006

[2] Viboud C Alonso WJ Simonsen L. Influenza in tropical regions. PLoS Med. (2006) 3:e89. 10.1371/journal.pmed.0030089 16509764 PMC1391975

[3] Yang R Yang J Wang L Xiao X Xia J. Contribution of local climate zones to the thermal environment and energy demand. Front Public Health. (2022) 10:992050. 10.3389/fpubh.2022.992050 36016886 PMC9395604

[4] Yang Z Ding Q Wang N Liu H. Distribution characteristics of health vulnerability and its influence factors in China. Scie Geographica Sinica. (2018) 38:135–42. 10.13249/j.cnki.sgs.2018.01.015

[5] Zhang Q Meng X Shi S Kan L Chen R Kan H. Overview of particulate air pollution and human health in China: evidence, challenges, and opportunities. Innovation. (2022) 3.:312 10.1016/j.xinn.2022.100312 36160941 PMC9490194

[6] Paules C Subbarao K. Influenza. Lancet. (2017) 390:697–708. 10.1016/S0140-6736(17)30129-0 28302313

[7] Tamerius J Nelson MI Zhou SZ Viboud C Miller MA Alonso WJ. Global influenza seasonality: reconciling patterns across temperate and tropical regions. Environ Health Perspect. (2011) 119:439–45. 10.1289/ehp.1002383 21097384 PMC3080923

[8] The Central People's Government of the People's Republic of China. Opinions of the State Council on the Implementation of Healthy China Action. (2019). Available online at: http://www.gov.cn/zhengce/content/2019-07/15/content_5409492.htm (accessed August 30, 2022).

[9] Cromer D Van Hoek AJ Jit M Edmunds WJ Fleming D Miller E. The burden of influenza in England by age and clinical risk group: a statistical analysis to inform vaccine policy. J Infect. (2014) 68:363–71. 10.1016/j.jinf.2013.11.013 24291062

[10] Czarkowski MP Hallmann-Szelinska E Staszewska E Bednarska K Kondratiuk K Brydak LB. Influenza in Poland in 2011-2012 and in 2011/2012 and 2012/2013 epidemic seasons. Przegl Epidemiol. (2014) 68:455–63. 25391010

[11] Tan E Hou H Bao H Teng X Zhang S Li B. Application of an autoregressive integrated moving average model for the prediction of influenza cases in China. Chin J Virol. (2017) 33:699–705. 10.13242/j.cnki.bingduxuebao.003221

[12] Fuhrmann C. The effects of weather and climate on the seasonality of influenza: what we know and what we need to know. Geography Compass. (2010) 4:718–30. 10.1111/j.1749-8198.2010.00343.x

[13] Clay K Lewis J Severnini E. Pollution, infectious disease, and mortality: evidence from the 1918 Spanish influenza pandemic. J Econ Hist. (2018) 78:1179–209. 10.1017/S002205071800058X

[14] Huang L Zhou L Chen J Chen K Liu Y Chen X. Acute effects of air pollution on influenza-like illness in Nanjing, China: a population-based study. Chemosphere. (2016) 147:180–7. 10.1016/j.chemosphere.2015.12.082 26766354

[15] Kalpazanov Y Kurchatova G Stamenova M. Air pollution and influenza epidemic in Sofia in 1972. Z Gesamte Hyg. (1975) 21:683–5. 1226919

[16] Liu X Li Y Qin G Zhu Y Li X Zhang J. Effects of air pollutants on occurrences of influenza-like illness and laboratory-confirmed influenza in Hefei, China. Int J Biometeorol. (2019) 63:51–60. 10.1007/s00484-018-1633-0 30382350

[17] Somayaji R Neradilek M Szpiro A Lofy K Goss C Jackson M. Effects of air pollution and environmental parameters on models estimating influenza-associated hospitalizations. C63 VIral Respiratory Infections. American Thoracic Society (2017). p. A6055–A.

[18] Xu Z Hu W Williams G Clements AC Kan H Tong S. Air pollution, temperature and pediatric influenza in Brisbane, Australia. Environ Int. (2013) 59:384–8. 10.1016/j.envint.2013.06.022 23911338

[19] Cao B Xu J Shu C Leng Y Shi X Su Y. Analysis on the outbreak of influenza epidemic characteristics and influencing factors in Heilongjiang province from 2013 to 2017. Chinese J Public Health Manag. (2018) 34:621–3+37. 10.19568/j.cnki.23-1318.2018.05.012

[20] Liao Q. Short-Term Impact of Air Pollution on Influenza-Like Illness in Yichang During 2014–2017. Wuhan: Huazhong University of Science and Technology. (2019).

[21] Li Y Dong T Jiang X Wang C Zhang Y Li Y. Chronic and low-level particulate matter exposure can sustainably mediate lung damage and alter CD4 T cells during acute lung injury. Mol Immunol. (2019) 112:51–8. 10.1016/j.molimm.2019.04.033 31078116

[22] Zhou J. Health effects of air pollution. Bull Chin Academy Sci. (2013) 28:371–7. 10.3969/j.issn.1000-3045.2013.03.011

[23] Mou F. Health effects of long-term exposure to low concentrations of sulfur dioxide on workers. Prevent Med Tribune. (2005) 11:298–9. 10.3969/j.issn.1672-9153.2005.03.0258280639

[24] Li H Yu W Liu Y. Health risk assessment of urban nitrogen dioxide, suspended particulate matter and sulfur dioxide. Foreign Med Sci(Section of Medgeography). (2007) 28:133–5+44. 10.3969/j.issn.1001-8883.2007.03.014

[25] Varon J Marik PE Fromm Jr RE Gueler A. Carbon monoxide poisoning: a review for clinicians. J Emerg Med. (1999) 17:87–93. 10.1016/S0736-4679(98)00128-0 9950394

[26] Li J Meng Z. Current progress in atmospheric environmental toxicology in China. Asian J Ecotoxicol. (2012) 7:133–9.

[27] Feng C Li J Sun W Zhang Y Wang Q. Impact of ambient fine particulate matter PM_2.5_ exposure on the risk of influenza-like-illness: a time-series analysis in Beijing, China. Environ Health. (2016) 15:1–12. 10.1186/s12940-016-0115-2 26864833 PMC4750357

[28] Ali ST Wu P Cauchemez S He D Fang VJ Cowling BJ. Ambient ozone and influenza transmissibility in Hong Kong. Eur Respiratory J. (2018) 51:18. 10.1183/13993003.00369-2018 29563172 PMC5948170

[29] Su W Wu X Geng X Zhao X Liu Q Liu T. The short-term effects of air pollutants on influenza-like illness in Jinan, China. BMC Public Health. (2019) 19:1–12. 10.1186/s12889-019-7607-2 31638933 PMC6805627

[30] Ni Y Szpiro AA Young MT Loftus CT Bush NR LeWinn KZ. Associations of pre-and postnatal air pollution exposures with child blood pressure and modification by maternal nutrition: a prospective study in the CANDLE cohort. Environ Health Perspect. (2021) 129:047004. 10.1289/EHP7486 33797937 PMC8043131

[31] Lee ES Kim J-Y Yoon Y-H Kim SB Kahng H Park J. A machine learning based determining the effects of air pollution and weather in respiratory disease patients visiting at emergency department using a national emergency department information system in Seoul, Korea. Emerg Med Int. (2022) 2022:1–20. 10.1155/2022/4462018 35154829 PMC8828357

[32] Toczylowski K Wietlicka-Piszcz M Grabowska M Sulik A. Cumulative effects of particulate matter pollution and meteorological variables on the risk of influenza-like illness. Viruses. (2021) 13:556. 10.3390/v13040556 33810283 PMC8065612

[33] Cai Y Xing Y Hu D. On sensitivity analysis. J Beijing Normal Univer (Nat Sci). (2008): 9–16. 10.3321/j.issn:0476-0301.2008.01.003

[34] Chen W Tu H Peng C Hou Y. Comment on sensitivity analysis methods for environmental models. Environ Sci. (2017) 38:4889–96. 10.13227/j.hjkx.201704121 29965437

[35] Cao Y Gao L Yuan L LI W. Analysis of potential evaporation and its sensitivity in Liaoning Province. Scientia Geographica Sinica. (2017) 37:1422–9. 10.13249/j.cnki.sgs.2017.09.015

[36] Koffi EN Bergamaschi P Alkama R Cescatti A. An observation-constrained assessment of the climate sensitivity and future trajectories of wetland methane emissions. Sci Advances. (2020) 6:eaay4444. 10.1126/sciadv.aay4444 32300649 PMC7148105

[37] Xi Q Li Z Luo C. Sensitivity analysis of AnnAGNPS model's hydrology and water quality parameters based on the perturbation analysis method. Environ Sci. (2014) 35:1773–80. 10.13227/j.hjkx.2014.05.019 25055665

[38] Yang Y Yu Y Li D Lu X. Concentration and health risk assessment of PCBs in E-waste dismantling field. China Environ Sci. (2012) 32:727–35. 10.3969/j.issn.1000-6923.2012.04.02427503630

[39] Silverman BW. Density Estimation for Statistics and Data Analysis. New York: Chapman and Hall (1986).

[40] Getis A Ord JK. The analysis of spatial association by use of distance statistics. Geogr Anal. (2010) 24:189–206. 10.1111/j.1538-4632.1992.tb00261.x

[41] Spearman C. The proof and measurement of association between two things. Am J Psychol. (1987) 100:441–71. 10.2307/1422689 3322052

[42] Deng J. Gray System Theory Tutorial. Wuhan: East China University of Science and Techinology Press (1990). 10.3321/j.issn:0375-5444.2005.02.007

[43] Liu Y Li R Song X. Grey associative analysis of regional urbanization and eco-environment coupling in China. Acta Geograph Sin. (2005) 60:237–47.

[44] Thomas M Joy AT. Elements of Information Theory. New Jersey, NJ: Wiley-Blackwel (2006).

[45] Thurstain-Goodwin M Unwin D. Defining and delineating the central areas of towns for statistical monitoring us ing continuous surface representation. Trans GIS. (2000) 4:305–17. 10.1111/1467-9671.00058

[46] Xu D Huang Z Huang R. The spatial effects of haze on tourism flows of Chinese cities: Empirical research based on the spatial panel econometric model. Acta Geograph Sin. (2019) 74:814–30. 10.11821/dlxb201904014

[47] Yang K Yang Y Zhu Y Li C Meng C. Social and economic drivers of PM_2.5_ and their spatial relationship in China. Geograp Res. (2016) 35:1051–60. 10.11821/dlyj201606005

[48] Han Y Zhou Z. Evaluation on ecosystem services in haze absorption by urban green land and its spatial pattern analysis in Xi'an. Geograph Res. (2015) 34:1247–58. 10.11821/dlyj201507005

[49] Liu H Fang C Huang J Zhu X Zhou Y Wang Z. The spatial-temporal characteristics and influencing factors of air pollution in Beijing-Tianjin-Hebei urban agglomeration. Acta Geograph Sin. (2018) 73:177–91. 10.11821/dlxb201801015

[50] Merbitz H Buttstädt M Michael S Dott W Schneider C. GIS-based identification of spatial variables enhancing heat and poor air quality in urban areas. Appl Geograp. (2012) 33:94–106. 10.1016/j.apgeog.2011.06.008

[51] Wang Z Fang C Xu G Pan Y. Spatial-temporal characteristics of the PM_2.5_ in China in 2014. Acta Geograph Sin. (2015) 70:1720–34. 10.11821/dlxb201511003

[52] Zhou L Zhou C Yang F Che L Wang B Sun D. Spatio-temporal evolution and the influencing factors of PM2. 5 in China between 2000 and 2015. J Geograph Sci. (2019) 29:253–70. 10.1007/s11442-019-1595-0

[53] Ma L Zhang X. The spatial effects of China's haze pollution and the impact from economic change and energy structure. Chin Indus Econo. (2014):19–31. 10.19581/j.cnki.ciejournal.2014.04.002

[54] Aman AT Wibawa T Kosasih H Asdie RH Safitri I Intansari US. Etiologies of severe acute respiratory infection (SARI) and misdiagnosis of influenza in Indonesia, 2013-2016. Influenza Other Respi Viruses. (2021) 15:34–44. 10.1111/irv.12781 32666619 PMC7405185

[55] Chen R Yin P Meng X Wang L Liu C Niu Y. Associations between ambient nitrogen dioxide and daily cause-specific mortality: evidence from 272 Chinese cities. Epidemiology. (2018) 29:482–9. 10.1097/EDE.0000000000000829 29621056

[56] Li T Zhang Y Wang J Xu D Yin Z Chen H. All-cause mortality risk associated with long-term exposure to ambient PM_2.5_ in China: a cohort study. Lancet Public Health. (2018) 3:e470–7. 10.1016/S2468-2667(18)30144-0 30314593

[57] Liang L Cai Y Barratt B Lyu B Chan Q Hansell AL. Associations between daily air quality and hospitalisations for acute exacerbation of chronic obstructive pulmonary disease in Beijing, 2013–17: an ecological analysis. Lancet Planetary Health. (2019) 3:e270–e9. 10.1016/S2542-5196(19)30085-3 31229002 PMC6610933

[58] Liu C Chen R Sera F Vicedo-Cabrera AM Guo Y Tong S. Ambient particulate air pollution and daily mortality in 652 cities. N Eng J Med. (2019) 381:705–15. 10.1056/NEJMc1913285 31433918 PMC7891185

[59] Pandey JS Kumar R Devotta S. Health risks of NO_2_, SPM and SO_2_ in Delhi (India). Atmos Environ. (2005) 39:6868–74. 10.1016/j.atmosenv.2005.08.00418316264

[60] Song Y Zhang Y Wang T Qian S Wang S. Spatio-temporal differentiation in the incidence of influenza and its relationship with air pollution in China from 2004 to 2017. Chin Geograph Sci. (2021) 31:815–28. 10.1007/s11769-021-1228-2 34580569 PMC8457542

[61] Tao Y Zhong L Huang X Lu S-E Li Y Dai L. Acute mortality effects of carbon monoxide in the pearl river delta of China. Sci Total Environ. (2011) 410:34–40. 10.1016/j.scitotenv.2011.09.004 21978618

[62] Yan M Liu Z Liu X Duan H Li T. Meta-analysis of the Chinese studies of the association between ambient ozone and mortality. Chemosphere. (2013) 93:899–905. 10.1016/j.chemosphere.2013.05.040 23786810

[63] Monamele GC Vernet M-A Nsaibirni RF Bigna JJR Kenmoe S Njankouo MR. Associations between meteorological parameters and influenza activity in a subtropical country: case of five sentinel sites in Yaounde-Cameroon. PLoS ONE. (2017) 12:e0186914. 10.1371/journal.pone.0186914 29088290 PMC5663393

[64] Li R Duan P Hu R Fan L. Prediction of new influenza cases based on ARIMA and SVM mixed models. J Yunnan Nationalities Univer:Natural Sci Edition. (2021) 31:103–10. 10.3969/j.issn.1672-8513.2022.01.016

[65] Yang C Yang H Guo S Wang Z Xu X Duan X. Alternative ozone metrics and daily mortality in Suzhou: the China Air Pollution and Health Effects Study (CAPES). Sci Total Environ. (2012) 426:83–9. 10.1016/j.scitotenv.2012.03.036 22521098

[66] Li X Xu J Wang W Liang J-J Deng Z-H Du J. Air pollutants and outpatient visits for influenza-like illness in Beijing, China. PeerJ. (2021) 9:e11397. 10.7717/peerj.11397 34141466 PMC8179240

[67] Wei G Zhang Z Ouyang X Shen Y Jiang S Liu B. Delineating the spatial-temporal variation of air pollution with urbanization in the Belt and Road Initiative area. Environ Impact Assess Rev. (2021) 91:106646. 10.1016/j.eiar.2021.106646

[68] Zhang D Zhou C He B. Spatial and temporal heterogeneity of urban land area and PM2.5 concentration in China. Urban Clim. (2022) 45:101268. 10.1016/j.uclim.2022.101268

